# Recent Advances in the Synthesis and Stabilization of Nickel and Nickel Oxide Nanoparticles: A Green Adeptness

**DOI:** 10.1155/2016/3512145

**Published:** 2016-06-19

**Authors:** Muhammad Imran Din, Aneela Rani

**Affiliations:** Institute of Chemistry, University of the Punjab, New Campus, Lahore 54590, Pakistan

## Abstract

Green protocols for the synthesis of nanoparticles have been attracting a lot of attention because they are eco-friendly, rapid, and cost-effective. Nickel and nickel oxide nanoparticles have been synthesized by green routes and characterized for impact of green chemistry on the properties and biological effects of nanoparticles in the last five years. Green synthesis, properties, and applications of nickel and nickel oxide nanoparticles have been reported in the literature. This review summarizes the synthesis of nickel and nickel oxide nanoparticles using different biological systems. This review also provides comparative overview of influence of chemical synthesis and green synthesis on structural properties of nickel and nickel oxide nanoparticles and their biological behavior. It concludes that green methods for synthesis of nickel and nickel oxide nanoparticles are better than chemical synthetic methods.

## 1. Introduction

Nanoparticles (NPs) are cluster of atoms having at least one dimension in the size range of 1–100 nm. Owing to their unique optical, magnetic, catalytic, and electrical properties, they have potential applications in various fields [1]. The physicochemical properties of NPs are different as compared to those of their bulk counterparts owing to the fact that surface area to volume ratio increases and quantum effects become dominant as the size decreases. The increase in surface area to volume ratio alters the mechanical, catalytic, and thermal properties of material [2].

In recent years there is an emerging interest to synthesize magnetic NPs of Fe, Co, and Ni due to their superior magnetic properties and potential uses in many fields including catalysis, memory storage devices, and sensors. In the field of medicine they are used for magnetically controlled drug delivery, magnetic resonance imaging, and hyperthermia treatment of cancer cells [3–5].

Many physical and chemical methods including coprecipitation [6], sol-gel [7], microemulsion [8], hydrothermal reaction [9], electrospray synthesis [10], and laser ablation [11] are used to synthesize NPs. These methods may produce well defined pure NPs but they have low productivity, high cytotoxicity, low antioxidant potential, and low antimicrobial activity and are not environmental friendly [12].

There is a great concern to search for environmentally benign methods which results in the development of bionanotechnology. Bionanotechnology synthesizes NPs by using biological systems including bacteria, fungi, yeast, plants, and naturally occurring small molecules such as vitamins, proteins, peptides, and reducing sugars [13–16].

The combination of biological principles (i.e., oxidation/reduction) by microbial enzymes or plant phytochemicals with physical and chemical approaches results in the synthesis of NPs with desired functions [17, 18]. Biological synthesis provides an environmental friendly, simple, inexpensive approach for synthesizing NPs with an added advantage of stabilizing the formed NPs as plant secondary metabolites besides acting as synthetic agents also act as capping agent. Moreover, NPs synthesized by using green chemistry have no or low cytotoxicity as compared to chemically synthesized NPs which makes them efficient carrier of drugs for* in vivo* drug delivery applications [19].

Herein, we review the work done in the field of green synthesis of Ni and NiO NPs and discuss the role of reaction parameters on the structural properties of formed Ni NPs. We also present a comparative overview of influence of chemical synthesis and green synthesis on the Ni and NiO NPs structural properties and biological properties.

## 2. Properties of Nanoparticles

The physical and chemical properties of NPs are function of their size and shape and are therefore different as compared to size independent constant physical properties of bulk material. This difference in properties at nanoscale is due to their large surface area which makes them highly reactive and quantum size effects which become dominant at nanoscale. Some of the size dependent properties of NPs are briefly described here:

(i) *Band Gap*. The band gap between the valence band and conduction band increases as the size of NPs decreases.
(ii) *Melting Point*. The melting point or phase transition temperature of NPs is low and this decrease becomes more pronounced when the particle size gets below 5 nm.
(iii) *Mechanical Properties*. The probability of defects is low at nanoscale, due to which their mechanical strength is high and they are characterized as highly tough and hard materials.
(iv) *Electrical Properties*. The electrical conductivity is influenced in 2 ways at nanoscale. It decreases because of large surface scattering while it may increase because of better ordering.
(v) *Optical Properties*. Color or optical properties of NPs are highly dependent on the size of particle. This change in color can be explained on the basis of shift of *λ*_SPR_ to higher wavelengths as the particle size increases in case of plasmonic NPs.
(vi) *Magnetic Properties*. Due to large surface energy of materials at nanoscale, ferromagnetism vanishes and shifts to supermagnetism.
(vii) *Catalytic Properties*. The catalytic efficiency of NPs is very high as compared to bulk material due to their large surface area [20].

## 3. Significance of Nickel Nanoparticles

Nickel NPs find potential applications in various fields including electronics, magnetism [21], energy technology [22], and biomedicines [23]. Due to their high reactivity, operational simplicity, and eco-friendly properties they are used to catalyze various organic reactions including chemoselective oxidative coupling of thiols [24], reduction of aldehydes and ketones [25], hydrogenation of olefins [26], synthesis of stilbenes from alcohol through Wittig-type olefination [27], and *α*-alkylation of methyl ketone [28]. They also catalyze certain inorganic reactions like decomposition of ammonia [29]. One of their recent applications is their role in the fabrication of carbon nanotubes (CNTs) [30].

They also find environmental applications in the field of adsorption of hazardous dye and inorganic pollutants and thus play a vital role in the cleanliness of environment [31]. Due to their good antibacterial and anti-inflammatory activities they are used in the field of biomedicine [19, 32].

They also show cytotoxicity against cancerous cells as is evident from the distortion of morphology of these cells after their treatment with Ni NPs [23, 33]. The biocompatibility of Ni NPs capped with biomolecules such as glucose is highly increased and these are used as biosensors and heat nonmediator for cancer hyperthermia [34].

## 4. Strategies for Nanoparticles Synthesis

Principally there are 2 approaches used to synthesize NPs including top-down approach and bottom-up approach.

### 4.1. Top-Down Approach for NPs Synthesis

This method comprises a set of synthetic technologies which synthesize NPs by removing certain parts from a bulk material substrate. The different methods for removal of parts from bulk materials may include chemical, electrochemical, and mechanical methods. The choice of a particular method is based on the material of bulk substrate and desired sizes of NPs. This technique however does not provide a full control on particle size. Top-down method is extended to obtain nanosized domains and coupled the mechanical removing techniques with electrochemical and chemical techniques [35].

### 4.2. Bottom-Up Approach for NPs Synthesis

This approach consists of set of synthetic technologies which synthesize larger and more complex systems by stacking materials on the top of a base substrate and maintaining good control over molecular structure. One of the basic requirements for this fabrication approach is that there must be strong adhesion forces between the surface layer and base substrate and for this purpose surfactants are added which increase the adhesion between surface layer and base substrate [35].

## 5. Mechanism for Biosynthesis of Metal and Metal Oxide Nanoparticles

The secondary metabolites of plants and microbial/fungal enzymes are responsible for the reduction of metal ions into metal atoms. The metal salts like nitrates, chlorides, oxides, and sulphates have high reduction potential due to attachment of metal with the chloride, oxide, and sulphide parts and their tendency to donate electrons. As a result of both these factors electronic density on the conjugative salts of metal increases. So metals in their ionic form can easily get detached from their anionic part and get reduced into stable form by using plant/microbial/fungal extract. The secondary metabolites of plant including alkaloids, flavonoids, polyphenols, and terpenoids act as chelator to metal ions and reduce them into zero-valent states. Mostly the –OH group of polyphenols and flavonoids develop coordination with metal ions, while in case of microbial mediated synthesis the reductase enzyme of bacterial or fungal cell wall donates electron for reduction of metal ions.

**Figure 1:**
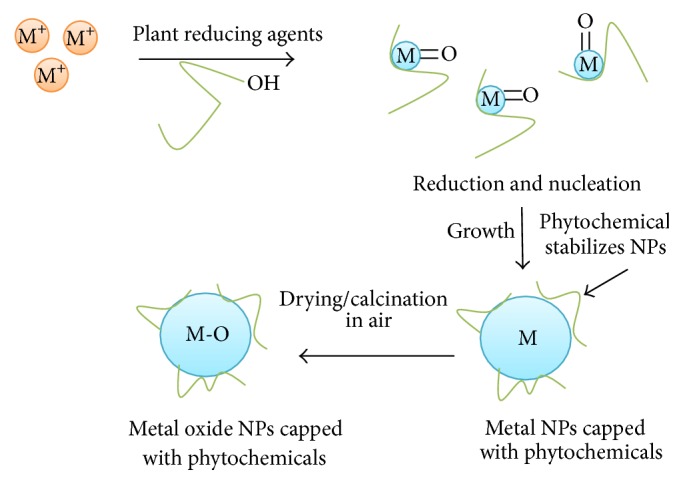
Mechanism of plant mediated synthesis of metal and metal oxide nanoparticles.

We can describe mechanism of plant mediated synthesis of metal and metal oxide NPs by considering the following three phases: (1) activation phase involves the reduction of metal ions and reduced metal atoms undergo nucleation; (2) growth phase involves the spontaneous coalescence of small adjacent NPs into larger size NPs, that is, Ostwald ripening (a process in which NPs are directly formed through heterogeneous nucleation and growth and further reduction of metal ion); this process enhances the thermodynamic stability of NPs; (3) termination phase decides the final shape of NPs. In case of metal oxide NPs the end product is air-dried or calcined in air to get final metal oxide NPs [36]. The schematic diagram for mechanism of NPs synthesis by plants is shown in Figure 1.

The mechanism of microbial mediated synthesis of metal and metal oxide NPs is also described by the following three phases: (1) the metal cation is trapped by bacterial or fungal cell wall due to electrostatic interactions between negatively charged cell wall and positively charged metal cation; (2) the cell wall then releases reductase enzyme which reduced the metal cations into metal atom; (3) these atoms then aggregate and form metal NPs. The formed NPs might be capped by the biomolecules of bacterium or fungi which prevent further aggregation of metal NPs and finally the formed NPs diffused out from cell wall [37].

**Figure 2:**
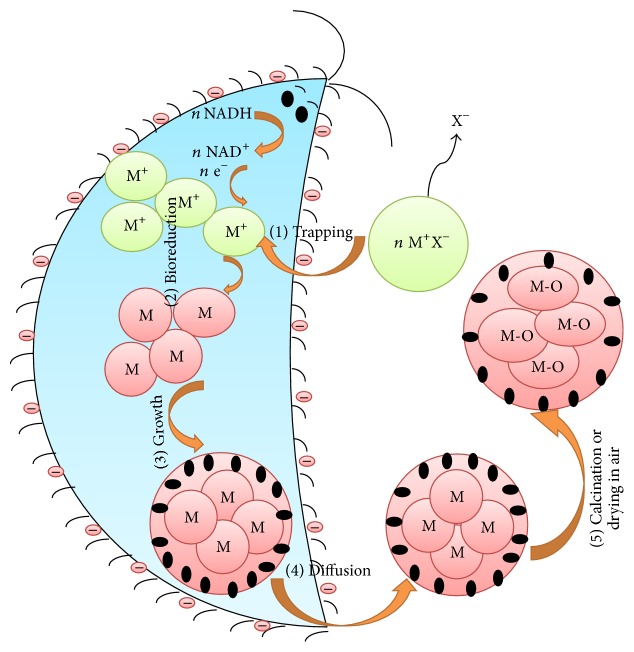
Mechanism of microbes mediated synthesis of metal and metal oxide nanoparticles.

In case of metal oxide NPs the end product is air-dried or calcined in air to get final metal oxide NPs. The schematic diagram for mechanism of NPs synthesis by plants is shown in Figure 2.

As the growth phase duration increases, aggregation of NPs occurs and nanohexahedrons, nanotubes, nanoprisms, and different kinds of irregularly shaped NPs are formed. The aggregation occurred because of stronger binding energy between 2 metal atoms as compared to atom-solvent binding energy. The aggregation of NPs is prevented somewhat by the secondary metabolites of plants and fungal/microbial biomolecules/enzymes which act as capping agent and stabilize the formed NPs [36]. Mallikarjuna and coworkers reported that the hydroxyl and carbonyl groups of amino acid residues or proteins can strongly adhere to metal NPs as capping agent and prevent them from aggregation [38].

**Figure 3:**
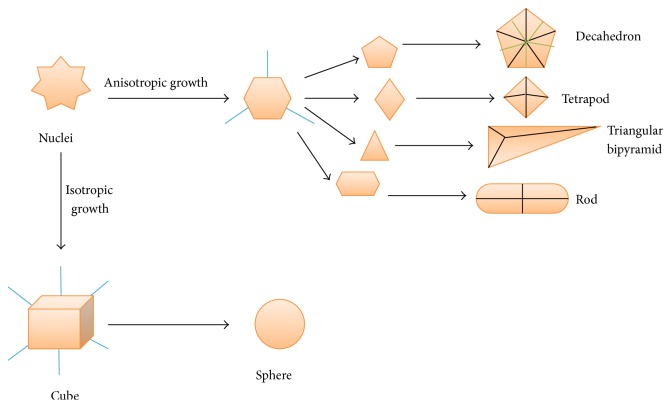
Growth of nanoparticles by different ways and their resulting geometrical shapes.

The crystal shape of metal and metal oxide NPs is a function of rate of growth in different crystallographic directions. The surface energy of crystal faces varies because the capping agents interact differently with different faces. This results in anisotropic growth of metal crystals, while in case of isotropic growth, rate of reaction is high and spherical shaped crystals are obtained. In case of higher rate of reaction, process of nucleation dominates over growth and vice versa. The size of NPs also increases when their growth is anisotropic. However, the dimensions of anisotropically growing nanomaterials (e.g., nanoprisms) can be controlled by adjusting the experimental conditions like pH, ratio of metal ion and reducing agent, irradiation time and its strength (in case of microwave heating), and reaction time.  Figure 3 shows the different shapes of NPs crystal that result from their isotropic and anisotropic growth [39].

## 6. Biosynthesis of Nickel Nanoparticles

Very few literature is available on the biological synthesis of Ni and NiO NPs as compared to chemical synthesis. The physical and chemical methods of NPs fabrication are accompanied by some disadvantages like high cost, complexity (involving multiple steps), use of harmful organic chemicals, and environmental pollution. So there is a great need to develop alternative eco-friendly and low cost fabrication methods for NPs. Nature has devised numerous processes for the fabrication of micro- and nanoscaled inorganic materials using naturally occurring biomolecules or microorganisms and plant extract as reducing agent. Green synthesis of NPs is a type of bottom-up technique where main reaction taking place is reduction/oxidation. There are 3 basic requirements for biosynthesis of NPs including (i) choice of proper solvent, (ii) choice of an eco-friendly reducing agent, and (iii) choice of nontoxic stabilizing agent for NPs. Thus by choosing proper solvent, surfactant and reductant biosynthesis produces NPs with controlled morphology without producing any toxic environmental pollutant [35].

In plant mediated synthesis of Ni NPs commonly extract of different parts of plants is used as a reducing agent, while in some other cases rather than using the extract either whole plant is grown on a metal substrate or an entire part of plant is soaked in metal solution. Here, in situ reduction of metal ions occurs and their morphology can also be controlled as porous parts of plants also act as biotemplate [40]. Some naturally occurring biomolecules such as glucose, sucrose, or plant secretions may also work as good reducing agent and stabilizing agents and thus are used to fabricate Ni NPs [16, 34, 41].

### 6.1. Biosynthesis of Ni Nanoparticles Using Plants

In recent years the fabrication of NPs using plants has fascinated the researchers because of its simple, cost effective, fast, and environmental friendly protocol [42]. Biosynthesis is a single step technique for synthesis of NPs which provides stable NPs of different morphologies. The rate of production is rapid compared to microorganisms based biosynthesis of NPs. Infra-red spectroscopy showed that secondary metabolites including terpenoids, flavones, pyrones, aldehydes, amides, and carboxylic acids derived from plant extracts are responsible for reduction of metal salts into their respective NPs [43]. Only few research articles are reported so far on the synthesis of Ni NPs using plants. Different parts of plants such as leaves and roots are used for synthesizing Ni NPs.

#### 6.1.1. Fabrication of Ni NPs Using Leaf Extract of Plants

Chen et al. [44] reported the synthesis of face-centered cubic Ni NPs by reducing aqueous solution of Ni(NO_3_)_2_ with aqueous extract of* Medicago sativa* (alfalfa). The typical synthetic method of Ni NPs involved the vigorous stirring of precursor solution with alfalfa solution at 60°C for 4 hours. The reaction was carried out at 60°C because at room temperature it is difficult to completely reduce Ni(II) into Ni(0). Then NPs solution was freeze-dried for 24 h in order to obtain Ni NPs powder.

Pandian and coworkers [31] synthesized Ni nanoparticles by using aqueous solution of Ni(NO_3_)_2_·6H_2_O as precursor and leaf extract of* Ocimum sanctum* as reducing agent as well as stabilizing agent. The Ni(II) ions were reduced into Ni(0) by hydrated electrons of* O. sanctum* aqueous leaf extract and Ni(0) nuclei were formed. These Ni(0) atoms then aggregate and Ni NPs were formed. A UV/Vis spectrum of the sample was recorded and peak centered at 395 nm corresponding to Ni NPs confirmed the formation of Ni NPs. The XRD pattern was recorded for Ni NPs confirming that Ni NPs have face centered cubic structure and average particle size was 30 nm as calculated by Debye-Scherrer equation. This particle size showed good agreement with that calculated by SEM (particle size was 15 and 36 nm) and by TEM (particle size was 12 and 36 nm).

These Ni NPs were then used to adsorb organic dyes including crystal violet (CV), 2-naphthol orange or Orange II (OR), eosin Y (EY), and anionic contaminants sulfate (SO_4_^2−^) and nitrate (NO_3_^−^) from aqueous solution. Their adsorption capacity was studied as a function of change in pH, Ni NPs dosage, contact time, and initial concentration of pollutants and dyes. The optimal conditions of pH, contact time, initial concentration of dyes, and inorganic pollutants for maximum adsorption capacity of Ni NPs are summarized in Table 1.

It was observed that adsorption of dye and inorganic pollutants increased by using large initial concentration of both Ni NPs and pollutants and increasing contact time of adsorbent and adsorbate.

#### 6.1.2. Fabrication of Nickel Nanoparticles Using Plants as Biotemplate

The leaves and petals of plants are porous and they serve as biotemplate for fabrication of Ni NPs. This method of Ni NPs synthesis is advantageous since it has good control on size of particle and Ni NPs formed are not prone to agglomeration. Kar and Ray [40] developed a green method for fabrication of metallic Ni NPs using petals of* Hibiscus rosa-sinensis* as biotemplate and reducing agent. The petals of hibiscus flowers are porous and beneath these pores tunnels are present which supply nutrients and moisture to whole parts of petal. These pores adsorbed NiCl_2_·6H_2_O which then subsequently reduced into Ni NPs by C_2_H_4_ which is one of the products obtained during pyrolysis of petals. The formation of Ni NPs was confirmed by the attraction of end product of reaction towards the magnet. The size of obtained Ni NPs was in the range of 10 nm–200 nm and was coated with the mesoporous carbon which prevents them from agglomeration. The formed Ni NPs were stable even after 20 days.

### 6.2. Fabrication of Bioconjugated Nickel Nanoparticles Using Naturally Occurring Biomolecules

The magnetic nanoparticles of nickel are prone to oxidation and therefore most of synthesis protocols utilize toxic and expensive organic media and hydrophobic capping agents to prevent agglomeration and surface oxidation of magnetic nanoparticles [45]. The stability of nickel nanoparticles in aqueous media is a challenge.

The naturally occurring biomolecules such as vitamins, reducing sugars, and plant secretions containing polyphenols are good antioxidants and they can potentially be applied to produce stable dispersions of nanoparticles in aqueous media where they act both as reductant and as capping agents. Moreover, the biocompatibility of formed NPs is also increased which thus increases their applications in the field of drug delivery.

Raj and Viswanathan [16] reported the synthesis of Ni NPs in ethanolic media by using sucrose as reducing agent and vegetable oil as capping agent. In order to establish suitable precursor and reducing agent several precursors (nickel nitrate, nickel acetate, and nickel chloride) and reducing agents (hydrazine, sodium borohydride, and sucrose) were investigated. The calculated heat of reaction showed that nickel nitrate was best precursor and sucrose was best reducing agent.

Bioconjugated NPs have wide range of applications in the biomedical field as NPs become more biocompatible for* in vivo* applications and often used as luminescence tagging, labeling, imaging, and drug delivery [46–48]. Some work has been done in the direction to synthesize bioconjugated Ni NPs in aqueous media using environmentally benign reducing and capping agent.

Vaseem et al. [34] synthesized highly water-stable bioconjugated glucose-capped nickel nanoparticles (G-Ni NPs) through aqueous solution process by using glucose both as a reducing agent and as a capping agent in the presence of liq. NH_3_. Due to their enhanced biocompatibility these were used as biosensors and heat nonmediator for cancer hyperthermia. The mechanism involved in the synthesis of G-Ni NPs was proposed as follows: firstly by the addition of liq. NH_3_ into Ni(NO_3_)·6H_2_O and glucose solution, green color ppt. of Ni(OH)_2_ were formed. By the further addition of liq. NH_3_ these ppt. dissolved and transparent green color solution is formed which was due to formation of [Ni(NH_3_)_4_]^2+^ complex. This complex was refluxed in ambient atmosphere at 80°C for 2 h. In the presence of liq. NH_3_ the aldehyde group of glucose oxidized into carboxylate ion and resulting free electrons reduced [Ni(NH_3_)_4_]^2+^ complex into Ni metal. Usually, glucose remains in open chain format; however in aqueous solution it transforms its structure into cyclic chair form. So, in addition to being acting as a reducing agent, here, it also acts as capping agent by developing complexation with Ni NPs through its five –OH groups. It is considered that at high pH condition surface of Ni NPs oxidizes to develop a negative charge (Ni-O^−^). Usually, in aqueous media metal NPs have negative surface charges; that is why it is considered that hydrogen bonding interactions develop between Ni NPs surface and glucose which facilitates capping of Ni NPs.

### 6.3. Characterization of Ni Nanoparticles

UV-Visible spectroscopy (UV-Vis) [31], Fourier transformation infrared spectroscopy (FTIR) [44], X-ray diffraction (XRD) [23, 40], scanning electron microscopy (SEM) [31], transmission electron microscopy (TEM) [44], zeta potential measurement [19], thermogravimetric analysis (TGA) [41], X-ray photoelectron spectrometry (XPS) [44], photoluminescence (PL) [41], energy-dispersive X-ray spectroscopy (EDS) [34], and atomic force microscope (AFM) [41] are literature reported fundamental characterization techniques for Ni NPs [31].

Ni NPs are plasmonic; that is, they show surface plasmon resonance (SPR) and absorption band in the range of 300 nm–400 nm is due to SPR of Ni. The phenomenon of SPR occurred because the metallic NPs physically absorbed light and as a result of this absorption conduction electrons of metal undergo coherent oscillation. This happened when the frequency of incident photon becomes equal to natural frequency of surface electrons; at this frequency amplitude of oscillation becomes maximum and this frequency is called SPR. The absorbance of light is measured with the help of UV/Vis spectrophotometer.

The shape of SPR band, its width, and its spectral position depend on the size of NPs and their size distribution. The SPR peak shows a red shift in position as the size of NPs increases, and blue shift is observed when particle size decreases. When the NPs are monodisperse in size distribution then shape of SPR peak is symmetrical and it became broad and split into two bands when size distribution became nonuniform [49].

Mamuru et al. synthesized Ni NPs using aqueous leaf extract of* Annona squamosa* as reducing agent and aqueous NiO solution as precursor salt at neutral pH. They monitored the formation of Ni NPs by visual color change of reaction mixture and by UV/Vis spectroscopy. The color of reaction mixture changes from light blue to dark brown indicating the formation of Ni NPs. Their formation was further confirmed by taking their UV/Vis spectrum in which absorbance maximum at 285 nm was assigned to SPR peak for Ni NPs. The plasmonic band had symmetrical shape which suggested that formed NPs were uniform and well-dispersed [50].

FTIR spectroscopy is used to investigate the biomolecules responsible for reduction of metal salt into metal NPs and their subsequent growth inactivation through the capping effect of these biomolecules. For this purpose, the FTIR spectrum of plant extract and metal NPs is recorded. The absorption band corresponding to biomolecules which are responsible for bioreduction should appear only in extract spectrum and should disappear in NPs spectrum. This helps not only in the identification of biomolecules responsible for bioreduction but in proposing the mechanism of reaction.

Mamuru and Jaji synthesized Ni NPs using NiCl_2_·6H_2_O as a salt precursor and leaf extract of* Moringa oleifera* as a reducing agent. The change in color of solution from light blue to dark reddish brown indicated the formation of Ni NPs which was further confirmed by observing their SPR peak at 297 nm. They tried to explore the biomolecules responsible for bioreduction of Ni(II) ions into Ni(0) by recording the FTIR spectrum of both leaf extracts of* M. oleifera* and Ni NPs. It was observed that IR band at 1636 cm^−1^ was the only one band that was present in* M. oleifera* spectrum and was absent in IR spectrum of Ni NPs. All other bands of* M. oleifera* were present in Ni NPs spectrum. This band was of amino aryl ketones, that is, anthraquinones, so it is possible that this is the biomolecule which was responsible for reduction. The presence of anthraquinone was confirmed by the appearance of cherish red color after the addition of 25% NH_3_ solution into leaf extract which is a confirmatory test for anthraquinones [49].

Another way to identify the biomolecules responsible for bioreduction is to record the FTIR spectrum of plant/microbial extract before and after bioreduction. The bands of those biomolecules which are responsible for bioreduction change their position in the spectrum recorded after bioreduction. In this regard, Chen and coworkers tried to explore the biomolecules of alfalfa grass which were responsible for the bioreduction of Ni(II) ions and stabilization of Ni(0) NPs by recording IR spectrum of extract before and after bioreduction. The bioreduction of Ni(II) was caused by flavonoids and reducing sugars of extract as indicated by the change in band position of C–O group of flavonoids and reducing sugar. The band was observed at longer wavelength after bioreduction as compared to the band recorded before bioreduction. The IR result suggesting flavonoids and reducing sugar as reductant was further supported by low amount of these biomolecules in extract measured after bioreduction as compared to their amount measured before bioreduction [44].

XRD is used for the identification, purity, and quantitative analysis of NPs. The phase of NPs is determined by recording the peaks at 2*θ* value; these peaks give the value of crystal planes for particular type of NPs. By comparing the position and intensity of these diffraction peaks with Joint Committee on Powder Diffraction Standard (JCPDS) card number (each type of metal has specific JCPDS card number) one can identify the NPs and their phase (spherical, wurtzite, etc.). The intensity of XRD spectrum peaks is function of particle crystallinity. When the NPs have good crystallinity then intense and sharp peaks are observed and vice versa. The NPs size can also be calculated using Scherrer equation; when particle size is large then XRD patterns become broad [51]:

(1) $$\begin{array}{c} D=\frac{0.98\lambda }{\beta \cos\theta }, \end{array}$$

where *D* is particle size, *λ* is wavelength (Cu K*α*), *β* is FWHM, and *θ* is diffraction angle.

Pandian et al. recorded the XRD spectrum for Ni NPs synthesized by reducing Ni(II) ions with leaf extract of* Ocimum sanctum*. They observed 5 distinct diffraction peaks in XRD spectrum at 2*θ* values 37.32°, 44.82°, 47.92°, 63.11°, and 72.97° and the peak at 2*θ* value 44.82° showed maximum intensity. Their resulting miller indices (111) and (200) affirmed that the NPs were face centered cubic (fcc) Ni. The average particle size calculated by Debye-Scherrer formula was found to be 30 nm [31].

SEM is used to study the surface morphology and composition of NPs by scanning the surface with high energy electron beam, produced by heated filament. Angajala et al. [52] synthesized Ni NPs by using the aqueous leaf extract of* Aegle marmelos* Correa (AmC) as a reducing agent and aqueous solution of NiCl_2_·6H_2_O as a precursor salt. The formation of Ni NPs was indicated by color change of solution from dark green to light green. The surface morphology of formed Ni NPs was investigated by employing SEM. The results indicated that NPs were polycrystalline in nature with average particle size of 80–100 nm having triangular shape. Their SEM images exhibited that NPs were capped by organic biomolecule layer which is derived from leaf extract of AmC having surface functional –OH groups that take part in bioreduction of Ni(II) ions into Ni(0) NPs, besides acting as stabilizing and capping agent. Within the agglomerates the NPs were not in direct contact and aggregation was seen only between the outer surfaces of organic material surrounding the Ni NPs.

TEM is used for the identification of details of internal composition of NPs including their shape, size, size distribution, and defects. Mariam et al. synthesized Ni NPs by using leaf extract of* Azadirachta indica *and NiO NPs using* Psidium guajava *leaf extract. Their morphology was determined by taking their TEM images. It was exhibited by their TEM images that both Ni and NiO NPs were spherical in shape and their size was <100 nm. Their size distribution was also narrow and NPs showed aggregation in order to reduce the total surface energy of system [23].

### 6.4. Effect of Reaction Parameters on Structural Properties of Ni NPs

The structural properties of Ni NPs such as their size and shape are function of reaction parameters including concentration of leaf extract, concentration of precursor salt, temperature, pH of medium, and reaction time. Chen et al. studied the effect of concentration of alfalfa extract on the size of Ni NPs. It was observed that not only particle size increased but also widening of size distribution occurred at high concentration of extract. This was due to the fact that by increasing the concentration of extract concentration of reducing agents increased for the same concentration of precursor salt and thus size of Ni(0) particles grew with more and more Ni(0) produced by bioreduction [44].

### 6.5. Impact of Green Synthesis on the Biological Behavior of Ni NPs

NPs synthesized by green routes are more biocompatible and nontoxic because in this route the reducing agent and stabilizing agents for NPs are plants or microbial reducing sugars and flavonoids which do not have cytotoxic effects. In this regard Sudhasree et al. [19] carried out comparative study between conventional chemical method using hydrazine as a reducing agent and biological method using* Desmodium gangeticum* (DG) root extract for Ni NPs synthesis. DG possesses antioxidant and antiapoptotic properties. They compared the antioxidant potential and cytotoxicity and antimicrobial activity of Ni NPs synthesized by both routes.

The antioxidant potential of Ni NPs was assayed by studying the scavenging assay of 2,2-diphenyl-1-picrylhydrazyl (DPHH) and superoxide radical. DPHH violet color in the presence of radical changes to yellow color on hydrogenation. The higher percentage scavenging was observed in case of Ni NPs synthesized by green route (NiGs) as compared to Ni NPs synthesized by chemical methods (NiCs). This was attributed to the presence of an additional moiety in NiGs which was phenolic compounds and the presence of phenolic compounds was confirmed by high superoxide scavenging activity of NGs (phenolic compounds possess O_2_^−^ scavenging activity) [19].

NPs have ability to cross physiological barriers and thus can enter and damage cells of living organisms. The cytotoxicity of both types of NPs was checked by both* in vitro* and* in vivo* methods. In both these methods the activity of lactate dehydrogenase (LDH) which was released from plasma membrane because of discharge from damaged cell as a result of NPs presence was monitored to check the cytotoxicity of Ni NPs. The* in vitro* assay was carried out on epithelial cell line and* in vivo* assay was carried out on LLC PK1 kidney cell lines of male rate. The results showed that NiGs are nontoxic as compared to NiCs as confirmed by the low activity of LDH (formation of formazon by LDH catalyzed reduction of tetrazolium salt) in case of NiGs nanoparticles [19].

**Figure 4:**
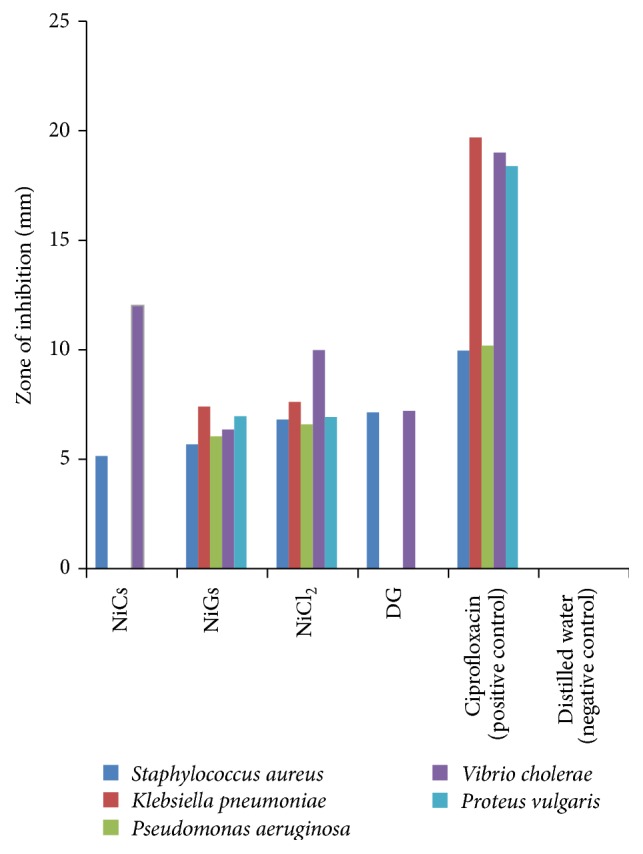
Comparison of antibacterial activities of NiGs, NiCs, NiCl_2_, DG, positive control, and negative control.

The antibacterial activity of NPs is a function of chemical composition, shape, concentration, photoactivation, and size of NPs. The antibacterial activity of NiGs and NiCs NPs, precursor salt, and reducing agent was assayed on Gram negative and Gram positive bacteria using agar gel diffusion method. Two control experiments, one positive control and one negative control, were also conducted to check antimicrobial activity. The large value for zone of inhibition in case of NiGs (Figure 4) confirmed that NiGs have high antibacterial activity as compared to that of NiCs, precursor salt, and DG extract [19]. Thus NiGs were nontoxic and possess higher stability and higher antioxidant and antimicrobial potential than chemically synthesized Ni NPs [19].

**Figure 5:**
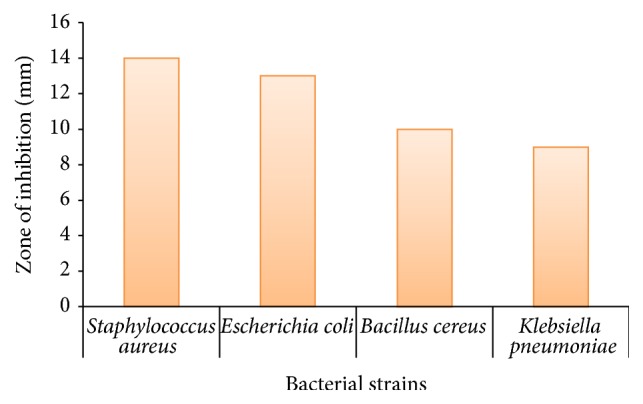
Comparison of effectiveness of Ni nanoparticles against different bacterial strains.

Helen and Rani [53] synthesized Ni NPs using aqueous solution of nickel sulphate and root tuber extract of* Dioscorea* (Elephant Yam) as reducing and capping agent. The color change of solution from blue to yellow indicated the formation of Ni NPs. The UV/Vis peak of Ni NPs was centered at 207 nm. The antibacterial activity of these Ni NPs was assayed by disc diffusion method against 4 bacterial strains including* Staphylococcus aureus* and* Bacillus cereus* (Gram positive) and* Klebsiella pneumonia* and* Escherichia coli* (Gram negative). The Ni NPs were most effective against* Staphylococcus aureus* and were least effective against* Klebsiella pneumonia* as indicated by their zone of inhibition. The result of effectiveness of Ni NPs against different microbes is shown in Figure 5.

Angajala and Radhakrishnan [32] synthesized Ni NPs by using aqueous leaf extract of* Aegle marmelos *Correa (AMC) and also checked their synergistic efficacy with *β*-sitosterol to induce anti-inflammation and compared their anti-inflammatory activity with the different leaf extracts of AMC by employing 2 tests, albumin denaturation assay and membrane stabilization test. *β*-Sitosterol is a plant sterol identified from the AMC aqueous leaf extract and it possesses anti-inflammatory properties. The –OH group of *β*-sitosterol develops bonding with AMC leaf extracts' synthesized Ni NPs surface. The intracellular inhibitory effect of Ni NPs associated *β*-sitosterol upon entering into inflammatory sites results because they develop association with receptor site by generating therapeutic effects and together assist the growth of peripheral blood lymphocytes. It was observed that the therapeutic efficacy of Ni NPs associated *β*-sitosterol was higher as compared to *β*-sitosterol of aqueous leaf extract. This was due to high surface area of Ni NPs as compared to aqueous leaf extract of AMC, which cause the *β*-sitosterol to remain on the surface of Ni NPs rather than in the interior of particle and therefore enhance its exposure to receptor sites in order to produce therapeutic effect.

Mariam et al. [23] synthesized Ni NPs using leaf extract of* Azadirachta indica* and NiO NPs using* Psidium guajava* leaf extract. The synthesized nanoparticles showed cytotoxicity against HT29 cell line (human colon adenocarcinoma). Two types of experiment were conducted on human colon adenocarcinoma cells; one of them was control experiment (untreated cells) and the other was test experiment in which cells were treated with Ni and NiO NPs. The cytotoxicity of Ni NPs and in turn the percentage viability of cancer cells were assayed by conducting a colorimetric assay, MMT assay (3-[4,5-dimethylthiazol-2-yl]-2,5-diphenyl tetrazolium bromide). MMT is a yellow tetrazol which can be reduced to insoluble crystals of purple formazan by the action of oxidoreductase enzymes of living cells. The insoluble crystals of formazan can be solubilized by adding dimethyl sulfoxide and absorbance of purple solution is quantified by recording spectra at certain wavelength (540 nm) using a spectrophotometer. The absorbance is directly proportional to number of living cells. The results of Mariam et al. [23] study showed that absorbance was decreased in cell culture treated with Ni NPs (concentration > 6.25 *μ*g/mL) as compared to that of untreated cells. These results were further supported by the morphological analysis of treated and controlled cells. The control cells showed smooth and regular surface with normal morphology, whereas Ni and NiO treated cells showed changes in cell morphology due to cell swelling and breaking as a result of apoptosis.

Nanoparticles are efficient drug carriers for both* in vitro* and* in vivo* applications. Chen and coworkers [33] checked the synergistic impact of Ni NPs with antitumor drug Verbascoside (VB) both* in vitro* and* in vivo* on the induction of apoptosis of K562 cancer cells. They conducted four types of experiments; one of them was control experiment in which cells were not treated and the other 3 experiments involved the treatment of cells with VB-Ni NPs, Ni NPs alone, and VB alone for 72 h, 48 h, and 24 h, respectively. The rate of apoptosis for* in vitro* drug and NPs treated cells was checked by identifying the characteristics feature of apoptotic nuclei like fragmented DNA and condensed chromosomes using fluorescence microscope. The result showed that rate of apoptosis was higher in VB-Ni NPs treated cells as compared to that of alone Ni and VB treated cells. The* in vivo* studies on the cancerous cells of female mice were conducted on the same line as was done for* in vitro* study, but here a magnet was also placed under mice skin near cancer cells. In this study, again rate of apoptosis was higher in VB-Ni NPs treated cells. These results suggested that Ni was transferred from VB-Ni into cancer cells through the action of magnetic field generated by the magnet placed near the cancer cells where it suppresses the growth of tumor cells and kills the tumor cells together with drug.

## 7. Nickel Oxide (NiO) Nanoparticles

Metal elements are capable of forming large diversity of compounds with oxygen (metal oxides). These metal oxides can be insulators, semiconductors, or conductors depending on their structural geometries which give rise to particular electronic structure. Metal oxides are widely used in the manufacturing of fuel cells, microelectronic circuits, piezoelectric devices, sensors, and corrosion resistant coatings and as catalysts. The metal oxide NPs possess distinct physical and chemical characteristics because of their smaller size and highly dense edge or corner surface sites. In any material, particle size affects the 3 most crucial groups of fundamental properties; first group is of structural properties, namely, cell parameters and lattice symmetry; second group is of electronic properties and the above mentioned two properties then induce changes in physical and chemical properties of materials. Among the metal oxide NPs, magnetic metal oxide NPs are gaining much interest because their properties can be modified according to their shape and size [54].

Recently, NiO NPs are studied widely because of their electrocatalysis, high chemical stability, superconductance characteristics, and electron transfer capability [55]. NiO is a p-type semiconductor metal oxide having a band gap ranging from 3.6 to 4.0 eV depending upon the nature of defects and their density. It is an antiferromagnetic material having Neel temperature *T*_N_ of ~523 K and besides a high isoelectric point of ~10.7, it also shows high ionization. The potential applications of NiO are in various areas like in water treatment, gas sensing, electrochemical performance, and antimicrobial activities. Numerous methods to fabricate NiO NPs have been reported in the literature which includes solvothermal [56], precipitation-calcination [57], chemical precipitation [58], microwave-assisted hydrothermal [59], and thermal decomposition [56, 60] methods. These methods involve ample reactants and starting materials, draggy procedures, and complex apparatus. Therefore, recently environmental benign green chemistry approach is used to synthesize NiO NPs.

### 7.1. Fabrication of NiO Nanoparticles by Using Plant Extract

Yuvakkumar and coworkers [61] synthesized NiO nanocrystals by utilizing nickel nitrate as precursor and rambutan peel waste as reducing and stabilizing agent and then checked their antibacterial activity by coating them on cotton fabric surface.

The probable mechanism for synthesis of NiO nanocrystals from rambutan peel extract is the formation of nickel-ellagate complex through ligation of phenolic hydroxyl group and ester oxygen atom of polyphenols with nickel at pH 5–7. The complex after calcination at 450°C decomposes and NiO nanocrystals are formed. The active components of rambutan are vitamins, polyphenols, flavonoids, and alkaloids which serve as antioxidants, antiviral, and radical scavengers. Among these active components the polyphenols such as ellagic acid, geranin, and corilagin are present in large amounts and serve as antioxidants.

**Figure 6:**
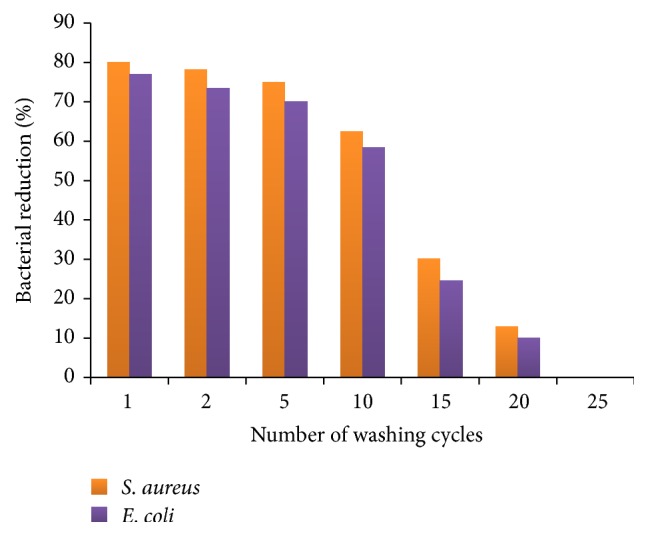
Effect of washing cycles on the antibacterial activity of NiO nanoparticles coated cotton fabric.

These NiO nanocrystals were then adsorbed on cotton fabric by pad-dry-cure and citric acid acts as crosslinker in this adsorption. The fabric untreated and treated with NiO nanocrystals was tested for antibacterial activity against* Staphylococcus aureus* (Gram positive bacteria) and* Escherichia coli* (Gram negative bacteria) by employing a disc diffusion method. The mechanism of antibacterial activity is described as follows: by the action of UV and visible light activation of NiO occurs which leads to formation of electron-hole pairs. By the hydrolysis and redox reactions hydrogen peroxide is produced which can enter cell membrane and kill bacteria. The antibacterial activity of fabric treated with NiO NPs was significant after 10 washes but after that the percentage of bacterial reduction was very low and after 20 washes this activity completely diminishes as shown in Figure 6.

Thema and coworkers synthesized single phase Bunsenite NiO NPs using* Agathosma betulina* leaves extract. The formed NPs were characterized for their surface/interface and volume room temperature characteristics by various analytical techniques. The photodiode behavior in the NIR spectral range for standard blade made film of pressed p-type NiO NPs onto n-type Si substrate was studied. The average size of NiO NPs was 26.7 ± 0.4 nm as calculated by their TEM and the crystalline aspects of these NPs were confirmed by observing many diffraction rings with strong diffraction spots using selective area electron diffraction (SAED). The elemental composition of formed NPs was confirmed by recording EDS spectrum which revealed the presence of Ni and O in the nanopowder. The presence of C and Cu elements was assigned copper grid and its carbon coating. The crystallographic analysis of formed NPs was carried out by recording their XRD profile. The obtained crystal planes were corresponding to cubic NiO phase which is also called Bunsenite phase. The average diameter of NiO NPs using Debye-Scherrer formula was within the range of 15.23–23.15 nm which was with close approximation with that size calculated by TEM.

NiO is a p-type semiconductor material having a weak absorption band in the visible region and an electrical resistivity on the basis of Ni cation vacancies concentration. There are no such Ni vacancies in the stoichiometric NiO; however at nanoscale there is probably a nonstoichiometric state of Ni depletion. The presence of these defects at nanoscale is attributed to the breakage of 3D symmetry at the NPs surface. Thus, any n-Si/p-type NiO NPs film heterojunction could show a photodiode behavior in the UV/Vis/NIR spectral range. The photodiode behavior of p-type NiO NPs deposited on n-type Si in the NIR spectral range was studied by applying Choi et al. photodiode experiment with these NPs. The normal incidence irradiation of photodiode was carried out employing a Hg lamp having a monochromator which covered the spectral range of 290–1100 nm. The p-NiO/n-Si reverse biased diode generated photocurrent at numerous wavelengths which covers the UV/Vis/NIR spectral range. By observing photo* I-V* profiles (photocurrent-voltage profiles) it is revealed that red illumination (748 and 650 nm) generated highest photoresponse followed by blue (430 nm) and zero current in dark. This can be explained in terms of the fact that the penetration depth of blue light in Si is smaller than red light so a smaller number of photocarriers are generated by blue light compared to that of red light which resulted in lower photocurrent in case of blue light illumination [55].

### 7.2. Fabrication of NiO Nanoparticles by Using Microbes

Microbes such as bacteria, yeast, and fungi are potential candidates that act as reductant and stabilizing agents for fabrication of nanoparticles. Although the rate of production of microbial synthesized nanoparticles is slower as compared to that of nanoparticles synthesized by using plant extracts they offer certain advantages like economic viability, simple scaling up, easy processing, and biomass handling.

#### 7.2.1. Preparation of Microbial Extract

The general method reported in the literature for the preparation of microbial extract involved the culturing of microbes on an appropriate broth medium followed by their incubation at suitable temperature and rpm on a rotary shaker for set number of days specific for microbes. Then culture is centrifuged at suitable rpm for specific time and supernatant is utilized for fabrication of Ni NPs. Incubation of fungi is carried out at 150 rpm and temperature of 25°C for 5 days [62], while bacteria are incubated at 200 rpm and temperature of 30°C for 120 hours [63]. The dead fungal biomass can be prepared by autoclaving the live biomass and then drying it at 50°C till it becomes crispy. The uniform size biomass particles are obtained after grinding the biomass [62].

#### 7.2.2. Fabrication of NiO NPs Using Fungi

Both living and dead fungal biomass can be used for fabrication of NPs. Use of dead fungal biomass is advantageous as it can be stored for longer period of time and does not require nutrients and growth media and also it has limited toxicity. One of the advantages of fungus mediated green synthesis of NPs is that large surface area can be recovered by optimum growth of mycelia. Moreover, the fungal biomass can tolerate metal toxicity by adsorbing the metal species on their cell wall which is composed of chitosan, chitin, amino group phosphates, glucan, lipids, sulphates, phospholipids and hydroxides, and so forth. These functional groups serve as binding sites for biosorption of metals [62].

One of the major dilemmas of this century is the increased contamination of natural aquatic bodies and sediments with toxic metals. During the last decade the introduction of Ni in the environment has been enhanced due to increased smelting and mining activities. The interaction between microbes and metals has been well established and the capability of microbes to accumulate and/extract metals is already used in bioremediation by the biosorption of toxic metals. However the mechanism behind this phenomenon has not been well established. Fungi are attractive candidates for biosorption of metals as they are capable of growing under extreme conditions of temperature, pH, and nutrient availability and at high concentration of metal [64].

Salvadori and coworkers [62] synthesized NiO NPs by using nickel chloride as precursor and dead, dried, and living biomass of filamentous fungus* Aspergillus aculeatus* (MIC = 2000 mg L^−1^) as reducing agent. Among the three types of fungal biomass used in this experiment maximum adsorption capacity and hence maximum resistance to metal toxicity were exhibited by dead biomass. The required doses of biomass and precursor solution were shaken under suitable reaction conditions and then the Ni(II) solution was separated from biomass by vacuum filtration using Millipore membrane. The obtained NiO NPs were analyzed through various techniques. The energy-dispersive X-ray spectroscopic analysis revealed the presence of proteins which may serve as capping agent on the NiO NPs surface in the form of film.

The same group of researchers synthesized NiO NPs by the biosorption of Ni(II) ions on the* Hypocrea lixii* fungus (MIC = 1473 mg L^−1^) living, dried, and dead biomass. The Ni(II) retention capacity of Ni for dead biomass was highest for dead biomass which indicated that dried and live biomass were susceptible to toxicity generated by high concentration of metal. The TEM and HRTEM micrographs of fraction of dead biomass loaded with NiO NPs revealed that NPs were present on the fungal cell wall both extracellularly and intracellularly through biosorption. The micrographs revealed that because of autoclaving process ultrastructural changes like shrinkage of cytoplasm were present both in NiO loaded dead biomass and in control sample. The average diameter of extra- and intracellular NiO fabricated NPs was 3.8 and 1.25 nm while their shape was spherical in both cases.

The mechanism for both above mentioned synthetic methods of NiO NPs by fungi is not fully elucidated. However, a 2-step mechanism was proposed; in first step adsorption of Ni(II) occurred with fungal cell wall through its amide group and then their reduction into metallic Ni by the enzymes of fungal cell wall. In the second step Ni was oxidized into NiO by the H_2_O and O_2_ present in the medium. The proteins/peptides of fungal cell wall acted as capping agent for formed NPs. This was confirmed by the EDX pattern of NiO NPs in which additional peaks for C, N, and O were present and further by their FTIR analysis in which band for N-H of amide II linkages of proteins/polypeptides at 1535 cm^−1^ was shifted to 1542 cm^−1^; this indicated the deformation of N-H group as it capped the NiO NPs [64].

Ullah and coworkers [65] synthesized NiO NPs by using nickel nitrate hex hydrate as precursor and* Rhizopus nigricans* fungus as reducing and stabilizing agent. The fungus was obtained from bread and its fine pieces were added into precursor solution. The pH of solution was adjusted by using 1 M NaOH solution. The solution after stirring for 5 hours was kept overnight and then filtered by using Whatman filter paper. This filtrate was then calcined at 500°C for 5 hours and the obtained sample was ground tiny particles. The characterization of NiO NPs revealed that the NPs are polydisperse with an average diameter of ~40 nm. Since these NPs are polydisperse, monodisperse NPs can be obtained by varying the experimental conditions like pH, temperature, and concentration of reductant and precursor.

#### 7.2.3. Fabrication of NiO NPs Using Bacteria

Nickel is widely used in steel, batteries, and electroplating industries and the Ni(II) ions discharged from these industries are carcinogenic so there is a need to reduce or remove their concentration in industrial effluents. The conventional effluent treatment methods are not efficient since they mostly introduce secondary pollutants in environment besides being costly. So there is a great concern to develop cost-effective and environmentally benign treatment methods which can either completely remove soluble Ni(II) ions from industrial discharge or convert them into insoluble separable form such as NiO which is insoluble in water.

Sathyavathi and coworkers [63] converted the soluble NiSO_4_ present in discharged effluents of electroplating industry into insoluble NiO by using cells of nickel resistant* Microbacterium* sp. MRS-1 which was isolated from effluent of nickel electroplating industry (effluent diluted with sodium chloride was plated on nutrient agar and incubated for bacterium growth at 30°C). The bioremediation of Ni(II) ions was achieved by incubating the bacterium with industrial effluent having Ni(II) of 2172 mg/L concentration at 30°C for 120 h at 200 rpm, followed by centrifugation of bacterial culture in order to remove cells of bacteria. The pale green precipitates were obtained at the bottom of flask which were then collected, washed, dried, and then characterized. The formation of metal oxide NPs is explained by efflux mechanism, which involves the extracellular fabrication of nanomaterials and by considering the role of metabolism dependent and biologically controlled processes which play major role in the nucleation and deposition of inorganic particles. The characterized precipitates were of NiO NPs having size in the range of 100 nm–50 nm with a flower like structure. Thus, this method provides a green route for the remediation of soluble toxic Ni(II) ions with an efficiency of 95% nickel removal.

### 7.3. Characterization of NiO Nanoparticles

UV-Visible spectroscopy (UV-Vis) [23], atomic force microscope (AFM), X-ray diffraction (XRD) [65], X-ray photoelectron spectrometry (XPS) [62], transmission electron microscopy (TEM) [61], scanning electron microscopy (SEM) [65], and energy-dispersive X-ray spectroscopy (EDS) [62] are fundamental techniques reported in literature for the characterization of NiO nanoparticles.

The NiO NPs synthesized extracellularly from* Microbacterium* sp. MRS-1 showed broad UV/Vis absorption band around 370–450 nm. The NiO NPs and functional groups of bacterial cell wall were studied by FTIR spectroscopy. The intense IR band at 580 cm^−1^ was assigned to Ni-O vibrations and present in both NIPE (NiO fabricated by the reaction of electroplating industrial effluent with MRS-1) and NiO (fabricated by the reaction of NiSO_4_ with MRS-1) and was absent in control (MRS-1 present in a solution without Ni(II) ions). The strong absorption peaks at 1024.34 cm^−1^ and 1064.74 cm^−1^ were assigned to amine group and these peaks suggested the role of amine group of bacterial cell wall in the adsorption of metal ions through hydrogen bonding and electrostatic interactions [63].

Salvadori et al. [62] fabricated film of NiO NPs coated on the surface of dead biomass of fungus and recorded the SEM images of dead biomass before and after coating of NiO NPs. It was observed that after binding of NiO NPs the surface of biomass becomes modified. The EDS spectra of biomass were also recorded before and after NiO formation and the spectra recorded after formation of NiO NPs gave a signal not only for Ni but also for C, N, and O which suggested the presence of proteins on the surface of NiO NPs. The proteins act as capping agents which stabilize the NiO NPs and are also responsible for the organization of NiO NPs on the surface of dead biomass of fungus in the form of film.

## 8. Conclusion

This paper provides an overview of green synthesis of nickel and nickel oxide nanoparticles by using plant extract, microbial extract, and naturally occurring biomolecules. Although all these green protocols for Ni and NiO nanoparticles synthesis have their own advantages and limitations use of plants extract as reductant is more beneficial as compared to microbial extract because of rapid rate of production of nanoparticles with former green reductant. However, all these green protocols for synthesis of Ni and NiO NPs were simple, efficient, and eco-friendly and did not require ample reactants, draggy procedures, and complex apparatus which were required in case of conventional chemical synthetic methods. The synthesis of nickel and nickel oxide nanoparticles by green chemistry is beneficial due to eco-friendliness, economic prospects, feasibility, enhanced biocompatibility, low cytotoxicity, and high antioxidant and high antimicrobial activity of formed nanoparticles. These features help in commercialization of Ni and NiO NPs in the fields of environmental cleaning and nanomedicine.

## 9. Future Perspectives

Ni and NiO nanoparticles with different structural properties and effective biological effects can be fabricated using new green protocols in coming days. The control over particle size and in turn size dependent properties of Ni and NiO NPs will open the new doors of their applications. There is a great potential of porous plant petals to act as soft biotemplate for Ni NPs which prevents them from agglomeration together holding good control over particle size. Only one plant has been reported as biotemplate for Ni NPs till date [40] so there is a great need to carry out more research in this direction. The work done so far on the green synthesis of Ni and NiO NPs is much less as compared to green protocols used in the synthesis of noble metal nanoparticles. Literature is available for selection of plants extracts as reducing agent for the synthesis of Ni NPs and NiO NPs [19, 23, 31, 32, 44, 49, 50, 52, 53, 55, 61]. The microbial mediated synthesis of Ni NPs is not reported yet but the future of this field is bright as reports are present on NiO NPs production using microbial reduction [63] and researcher can synthesize Ni NPs with some modifications in the protocols used for NiO NPs production through this route. Despite much work on plant and microbial mediated synthesis of NPs in this decade, the exact mechanism for their synthesis is not fully known and this is the major barrier in commercialization of these protocols. So, in order to scale up these protocols to industrial level the focus of a green chemist should not be only to synthesize NPs but also to fully explore the chemistry involved in synthesis.

## Figures and Tables

**Table 1:** Optimal reaction parameter conditions for maximum percentage removal of dyes and inorganic pollutants.

Adsorbate	pH	Contact time (min)	Initial conc. of dyes and pollutants (mg/L)
CV	8	40	40
EY	3	20	20
OR	3	30	30
NO_3_^−^	7	10	10
SO_4_^2−^	7	10	10
